# Lactoferrin Prevents Hepatic Injury and Fibrosis via the Inhibition of NF-κB Signaling in a Rat Non-Alcoholic Steatohepatitis Model

**DOI:** 10.3390/nu14010042

**Published:** 2021-12-23

**Authors:** Yoshinaga Aoyama, Aya Naiki-Ito, Kuang Xiaochen, Masayuki Komura, Hiroyuki Kato, Yuko Nagayasu, Shingo Inaguma, Hiroyuki Tsuda, Mamoru Tomita, Yoichi Matsuo, Shuji Takiguchi, Satoru Takahashi

**Affiliations:** 1 Department of Experimental Pathology and Tumor Biology, Nagoya City University Graduate School of Medical Sciences, 1-Kawasumi, Mizuho-cho, Mizuho-ku, Nagoya 467-8601, Japan; yoshinaga52@gmail.com (Y.A.); kuangxiaochen094@hotmail.com (K.X.); komura@med.nagoya-cu.ac.jp (M.K.); h.kato@med.nagoya-cu.ac.jp (H.K.); nagap1113@gmail.com (Y.N.); inaguma@med.nagoya-cu.ac.jp (S.I.); sattak@med.nagoya-cu.ac.jp (S.T.); 2 Department of Gastroenterological Surgery, Nagoya City University Graduate School of Medical Sciences, 1-Kawasumi, Mizuho-cho, Mizuho-ku, Nagoya 467-8601, Japan; matsuo@med.nagoya-cu.ac.jp (Y.M.); takiguch@med.nagoya-cu.ac.jp (S.T.); 3 Nanotoxicology Project, Nagoya City University, Nagoya 467-8603, Japan; htsuda@phar.nagoya-cu.ac.jp; 4 Dairy Techno Inc., Tokyo 105-0014, Japan; m-tomita@dairytechno.co.jp

**Keywords:** NASH, lactoferrin, fibrosis, hepatocarcinogenesis, connexin

## Abstract

Non-alcoholic steatohepatitis (NASH) can cause liver cirrhosis and hepatocellular carcinoma (HCC), with cases increasing worldwide. To reduce the incidence of liver cirrhosis and HCC, NASH is targeted for the development of treatments, along with viral hepatitis and alcoholic hepatitis. Lactoferrin (LF) has antioxidant, anti-cancer, and anti-inflammatory activities. However, whether LF affects NASH and fibrosis remains unelucidated. We aimed to clarify the chemopreventive effect of LF on NASH progression. We used a NASH model with metabolic syndrome established using connexin 32 (Cx32) dominant negative transgenic (Cx32ΔTg) rats. Cx32ΔTg rats (7 weeks old) were fed a high-fat diet and intraperitoneally injected with dimethylnitrosamine (DMN). Rats were divided into three groups for LF treatment at 0, 100, or 500 mg/kg/day for 17 weeks. Lactoferrin significantly protected steatosis and lobular inflammation in Cx32ΔTg rat livers and attenuated bridging fibrosis or liver cirrhosis induced by DMN. By quantitative RT–PCR, LF significantly down-regulated inflammatory (*Tnf-α*, *Il-6*, *Il-18*, and *Il-1β*) and fibrosis-related (*Tgf-β1*, *Timp2*, and *Col1a1*) cytokine mRNAs. Phosphorylated nuclear factor (NF)-κB protein decreased in response to LF, while phosphorylated JNK protein was unaffected. These results indicate that LF might act as a chemopreventive agent to prevent hepatic injury, inflammation, and fibrosis in NASH via NF-κB inactivation.

## 1. Introduction

The development of non-alcoholic fatty liver disease (NAFLD) is associated with obesity and disorders of lipid metabolism in patients with metabolic syndrome. With a global increase in recent years of the obese population, the number of cases of NAFLD has also increased [1,2]. A global meta-analysis describes the prevalence of NAFLD worldwide, which is approximately 25%, with the highest rates of 31% and 32% occurring in South America and the Middle East, respectively [3]. The concept of NAFLD is a broad spectrum of disease, ranging from simple steatosis without inflammation to non-alcoholic steatohepatitis (NASH) with chronic progressive inflammation and fibrosis. Continuous inflammation produces abundant inflammatory cytokines and accumulates reactive oxygen species (ROS) in the liver, leading to fibrosis. Once fibrosis develops to bridge cirrhosis, they are irreversible and can develop into hepatocellular carcinoma (HCC), as occurs in various chronic liver diseases, such as alcohol-induced injury and viral hepatitis [1,4].

Connexin (Cx) is a component of gap junctions, which exist between cells and is responsible for the transfer of small molecules less than 1 kDa, such as second messengers, ions, and cell metabolites [5,6]. This cellular interaction is called gap junctional intercellular communication (GJIC) and contributes to maintain tissue homeostasis and control cell growth and differentiation [7,8]. Within the liver, Cx32 exists as a major gap junction protein of hepatocytes [9,10]. In particular, decreased expression of Cx32 is followed by the continued progression of chronic liver diseases, such as liver cirrhosis and HCC [11].

We previously assessed the function of Cx32 in liver diseases via the establishment of Cx32 dominant negative transgenic (Cx32ΔTg) rats with a dominant negative mutant of Cx32 controlled by an albumin (Alb) promoter [12]. Cx32ΔTg rats showed greatly decreased Cx32 expression localized at the membrane and depressed GJIC capacity in their hepatocytes, as well as a high susceptibility to chemical-induced hepatocarcinogenesis compared to wild-type (Wt) rats [13,14]. Cx32 is involved in not only carcinogenesis but also NASH. There was no difference in susceptibilities to hepatotoxicity and hepatocarcinogenesis in the Cx32ΔTg as compared to Wt rats in basal diet feeding without any chemical treatment [14]. However, dysfunction of Cx32 in Cx32ΔTg rats exacerbated hepatocyte injury, steatohepatitis, and fibrosis due to increased ROS levels in the NASH induced by the methionine-choline deficient diet (MCDD) [10]. The acceleration of NASH development by Cx32 inactivation was also observed in another model that was induced by a combination of a high-fat diet (HFD) plus dimethylnitrosamine (DMN) in Cx32ΔTg rats [15]. Comparing the two models, nuclear factor (NF)-κB was commonly activated with the up-regulation of inflammatory cytokines, such as tumor necrosis factor (TNF)-α and transforming growth factor (TGF)-β1 in NASH induced by Cx32 dysfunction. Therefore, NF-κB is one of the key contributors to the progression of NASH.

Lactoferrin (LF) is an 80-kDa iron-binding glycoprotein found in all exocrine fluids, including tears, sweat, and saliva, and is especially abundant in milk. It was first isolated and purified in 1960 and was involved in the promotion of iron absorption and lipid metabolism. LF has various physiological functions, including anti-bacterial, anti-fungal, anti-viral, anti-oxidant, anti-cancer, anti-inflammatory effects, which have been reported. We focused on LF as a suppressor of inflammation. A previous study indicated that bovine LF inhibited chronic inflammation in the lungs in a mouse cystic fibrosis model. Tanaka et al. reported that bovine LF improved colitis in a dextran sulfate sodium-induced colitis model in rats and mice due to a reduction in the inflammation level by LF correlated with a decrease in proinflammatory cytokines, such as TNF-α, IL-1β, and interleukin (IL)-6 [16,17]. With regard to the liver, decreased IL-1β by LF leads to the inhibition of carbon tetrachloride-induced hepatitis in a rat model [18]. Another report suggests that LF reduced the expression of TGF-β1, IL-1β, and TNF-α and suppressed liver fibrosis in a rat systemic lupus erythematosus model [19]. The anti-tumor abilities of LF have also been described in various cancer cell lines, such as those of the breast [20], stomach [21], head, and neck [22]. A randomized placebo-controlled clinical trial indicated that the growth of colorectal adenomatous polyp was significantly retarded by intake of 3 g LF without any adverse events related to the intervention [23]. However, the effects of LF on hepatotoxicity, as well as fibrosis and carcinogenesis on NASH, have not been clearly established as yet.

In this study, we aimed to determine the chemopreventive effect of dietary LF on NASH development and hepatocarcinogenesis using a Cx32ΔTg–HFD–DMN NASH model.

## 2. Materials and Methods

### 2.1. Chemicals

A HFD (HFD-60) was bought from Oriental BioService, Inc. (Kyoto, Japan). DMN was supplied by Tokyo Kasei Kogyo Co. Ltd. (Tokyo, Japan). Bovine LF was provided by the Dairy Techno Inc. (Tokyo, Japan).

### 2.2. Development and Screening of Transgenic Rats

Cx32ΔTg rats were bred and screened as previously described [12]. Rats were housed in cages containing hardwood chips under specific pathogen-free conditions at 22 ± 2 °C and 50% humidity using a 12 h light/12 h dark cycle. Rats ate food and tap water that were available ad libitum. Protocols for animal experiments were approved by the Institutional Animal Care and Use Committee of Nagoya City University School of Medical Sciences (no. 19-025, approved on 24 September 2019).

### 2.3. Animal Treatments and Biochemical Analysis

A total of 48 male Cx32ΔTg rats (7 weeks old) ate a HFD for 17 weeks. After 5 weeks, DMN was injected intraperitoneally six times once every 2 weeks. DMN was used at 15 mg/kg (injections 1 and 2), 10 mg/kg (injections 3 and 4), and 5 mg/kg (injections 5 and 6). Rats were randomly divided into three groups (*n* = 16 each). One group of rats received tap water (Control), and the other two groups of rats continuously received either 100 or 500 mg/kg/day LF (LF100 or LF500) in drinking water for 17 weeks. During animal experiments, one rat in the control group unexpectedly died at week 10. Therefore, we analyzed 47 rats in total (Control: 15 rats, LF100: 16 rats, and LF500: 16 rats) once the experiment was completed. All rats were sacrificed under deep anesthesia, and samples of blood were taken from the abdominal aorta. Total adipose tissues around spermatic ducts were weighed to assess visceral fat.

The serum levels of Alb, total protein, alkaline phosphatase, aspartate aminotransferase (AST), alanine aminotransferase, high-density lipoprotein cholesterol, low-density lipoprotein cholesterol (LDL-C), total cholesterol, and glucose were assessed and measured at the DIMS Institute of Medical Science, Inc. (Aichi, Japan).

### 2.4. Histology of NASH

Rat livers were surgically excised and sliced into 3–4 mm thick sections. After fixing with 10% buffered formalin, sections were embedded in paraffin for histological evaluation (thickness 2–3 μm). Histological sections were stained with Azan or hematoxylin and eosin (H&E), as well as immunohistochemically stained with antibody against α-smooth muscle actin (α-SMA; Dako, Tokyo, Japan). Steatohepatitis and fibrosis were evaluated using a non-alcoholic fatty liver disease activity score (NAS), as previously described in detail [10,24]. The NAS and fibrosis scores were evaluated by three very experienced pathologists (A.N.-I., M.K., and S.Takahashi).

### 2.5. Evaluation of Preneoplastic Foci in the Liver

The glutathione S-transferase placental form (GST-P) was immunohistochemically stained, as previously described [25]. Averages of GST-P–positive foci that were >80 μm in diameter in the entire liver section were evaluated using an image analyzer (Keyence, Osaka, Japan).

### 2.6. Western Blotting

Protein samples were extracted from frozen liver tissues using radioimmunoprecipitation buffer (Thermo Fisher Scientific, Rockford, IL, USA) with added protease and phosphatase inhibitors (Thermo Fisher Scientific). The protein concentration of samples was quantified by a Bradford assay. Protein samples (30 μg per lane) were separated in 12% polyacrylamide gels and transferred onto nitrocellulose membranes (Hybond-ECL; GE Healthcare UK Ltd., Buckinghamshire, UK). Membranes were probed with primary antibodies against: Cdc42, IκB-α, NF-κB, phosphorylated (p) NF-κB (Ser536), Mkk4, pMkk4 (Thr261), Jnk, pJnk (Thr183/Tyr185) (Cell Signaling Technology, Danvers, MA, USA), and β-actin (Sigma-Aldrich, St. Louis, MI, USA). Anti–β-actin was used at a 1:5000 dilution, and all other antibodies were used at 1:1000. ImageJ software, ver.1.52 (National Cancer Institute, Bethesda, MD, USA), was used to quantify bands from blots.

### 2.7. Quantitative Reverse Transcription PCR

RNA samples were extracted, and quantitative reverse transcription (qRT)–PCR was performed, as previously described [15]. Phenol–chloroform was used to isolate total RNA from liver tissue (Isogen, Nippon Gene Co., Ltd., Tokyo, Japan) and then converted to cDNA with Moloney murine leukemia virus reverse transcriptase (Takara, Otsu, Japan). Quantitative reverse transcription was performed using an AriaMx Real-Time PCR system (g8830a, Agilent, Santa Clara, CA, USA). The sequences of primers used in this study were provided in a previous study [15].

### 2.8. Selection of a Candidate Reference Gene

In order to select a reference gene that is stably expressed and has low variability in the present experiment system, the stability of the five candidate housekeeping genes (Table 1) was validated using NormFinder (MOMA, Aarhus, Denmark). The relative quantification for qRT-PCR was performed by standard curve method.

### 2.9. Statistical Analysis

Data are presented as the mean ± standard deviation (SD), and one-way ANOVA and Tukey multiple comparison tests were used to compare differences between groups using the software package, Graph Pad Prism 8 (GraphPad Software, Inc., La Jolla, CA, USA). *p* < 0.05 was considered significant.

## 3. Results

### 3.1. LF Prevents Steatohepatitis and Fibrosis in Cx32ΔTg Rats

We initially investigated the safety and chemopreventive effect of LF on NASH in a Cx32ΔTg–HFD–DMN rat NASH model. The dosage of LF in previous clinical studies was to reflect the selection of dosages in the present study [23]. A significant difference in body weights between control and LF-treated groups was not found. A dose-dependent change in organ weights was not noted, although liver weights were significantly increased in the LF100 compared to control group (Table 2).

**Figure 1:**
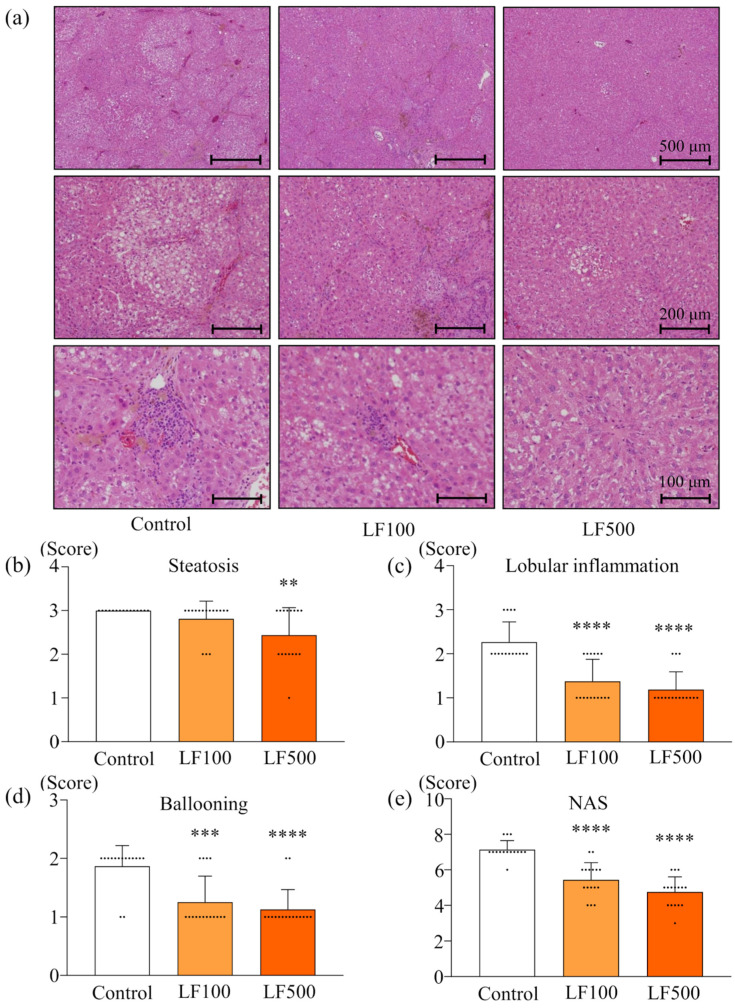
Preventive effect of lactoferrin on nonalcoholic steatohepatitis in rats. Connexin 32 dominant negative transgenic (Cx32ΔTg) rats were fed a high-fat diet (HFD), given an intraperitoneal injection of dimethylnitrosamine (DMN), and treated with lactoferrin (LF) for 17 weeks. (**a**) Representative histological findings of hematoxylin and eosin (H&E) stains in liver sections taken from Control, LF 100 mg/kg/day (LF100) or LF 500 mg/kg/day (LF500) rat groups. (**b**–**e**) Histopathological analysis of non-alcoholic steatohepatitis (NASH) was evaluated by severity scores for (**b**) steatosis, (**c**) lobular inflammation, (**d**) hepatocellular ballooning, and (**e**) a non-alcoholic fatty liver disease activity score (NAS). Data is shown as the mean ± SD, *n* = 15–16 per group, ** *p* < 0.01, *** *p* < 0.001, **** *p* < 0.0001 compared to the Control group.

**Figure 2:**
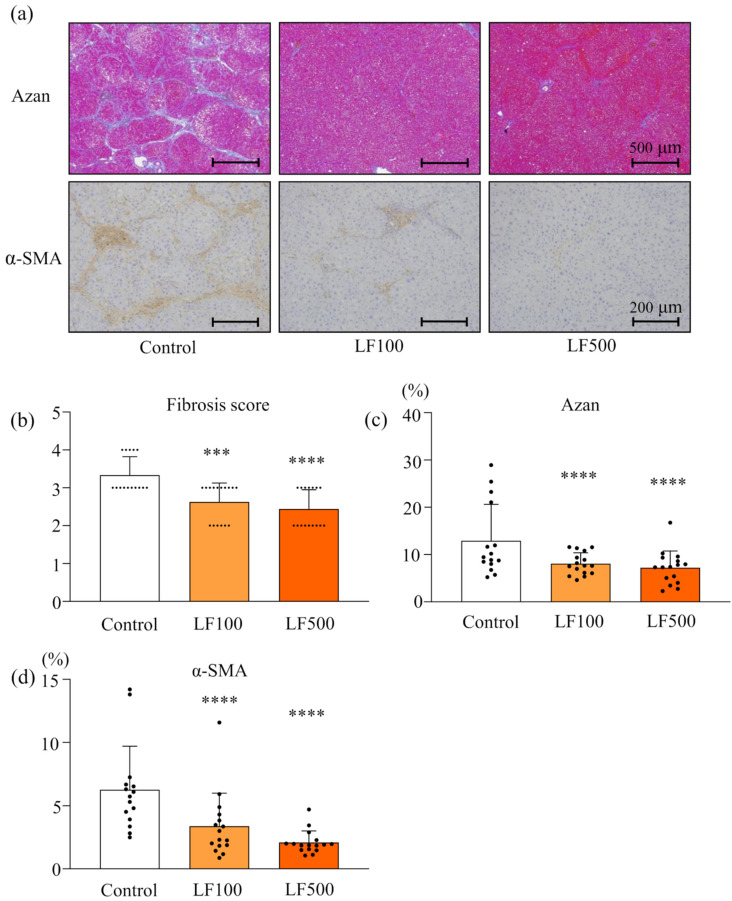
Attenuation effect of lactoferrin on fibrosis in rat nonalcoholic steatohepatitis. Connexin 32 dominant negative transgenic (Cx32ΔTg) rats were fed a high-fat diet (HFD), given an intraperitoneal injection of dimethylnitrosamine (DMN), and treated with lactoferrin (LF) for 17 weeks. (**a**) Azan staining (upper panels) and α-smooth muscle actin (α-SMA; lower panels) immunohistochemical stains of liver sections from Control, LF 100 mg/kg/day (LF100), or LF 500 mg/kg/day (LF500) rat groups. (**b**) Azan staining was used to evaluate the fibrosis score and (**c**) percentage of fibrosis area. (**d**) α-SMA–positive area. Data is shown as the mean ± SD, *n* = 15–16 per group, *** *p* < 0.001, **** *p* < 0.0001 compared to the Control group.

Histological observation by H&E staining also indicated that LF did not induce any changes in kidneys. In the liver, treatments of a HFD and DMN induced diffuse deposits of fat droplets with hepatocellular ballooning and neutrophil infiltration in the lobule (Figure 1a). Lactoferrin treatment significantly reduced fat deposition, lobular inflammation, and ballooning injury of hepatocytes in a dose-dependent manner (Figure 1a–d and Table S1), resulting in decreased NAS (Figure 1e and Table S1). Bridging fibrosis and activated hepatic stellate cells (HSC) were visualized by Azan staining and α-SMA immunohistochemical staining, respectively, in a Cx32ΔTg–HFD–DMN rat NASH model (Figure 2a). The histological fibrosis score, percentages of the Azan-positive area (collagen), and percentages of the α-SMA-positive area (activated HSCs) were significantly reduced by LF in the NASH model (Figure 2a–d and Table S1).

Biochemical analysis of serum indicated that the level of AST, T-chol, and LDL-C in the LF–treated groups was lower than that in the control group and the level of glucose of the LF100 was significantly higher than that of the control group. However, there was no dose-dependent change in serum hepatic enzymes, proteins, glucose, or lipids (Table 3). These results indicated that LF administration prevented the development of steatohepatitis and fibrosis without any adverse effects observed in a rat NASH model.

### 3.2. LF Tends to Decrease the Induction of Preneoplastic Lesions in Cx32ΔTg Rats

**Figure 3:**
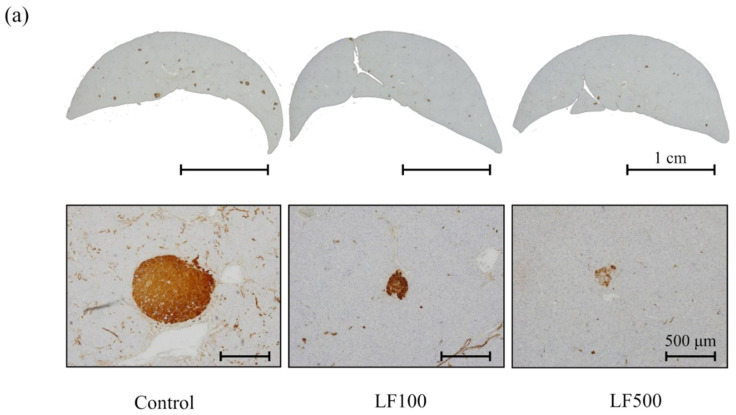

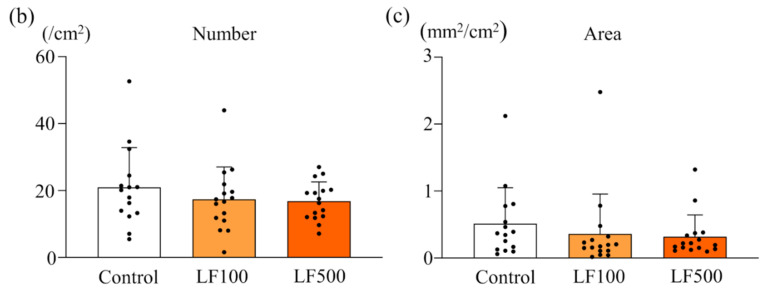
Effect of lactoferrin on hepatocarcinogenesis in rat nonalcoholic steatohepatitis. Connexin 32 dominant negative transgenic (Cx32ΔTg) rats were fed a high-fat diet (HFD), given an intraperitoneal injection of dimethylnitrosamine (DMN), and treated with lactoferrin (LF) for 17 weeks. (**a**) Liver sections showing representative foci positive for glutathione S-transferase placental form (GST-P) from Control, LF 100 mg/kg/day (LF100), or LF 500 mg/kg/day (LF500) rat groups. (**b**) The number and (**c**) area of GST-P–positive hepatic foci. Data is shown as the mean ± SD, *n* = 15–16 per group.

To explore the effect of LF on carcinogenic potential during the development of NASH, the formation of preneoplastic hepatic foci, namely GST-P–positive foci, was quantitated by immunohostochemistry. A combination of HFD and DMN treatment increased both the number and area of GST-P–positive foci in Cx32ΔTg rats, although the carcinogenic potential was weaker than that induced by MCDD plus diethylnitrosamine (DEN; Figure 3a–c) [10,15]. In contrast, both the number and area of GST-P–positive lesions tended to be decreased by LF intake (Figure 3a–c and Table S1). In accordance with these results, LF may have the potential to reduce hepatocarcinogenesis in NASH.

### 3.3. LF Down-Regulates mRNA Expression of Inflammatory Cytokines in Cx32ΔTg Rats

**Figure 4:**
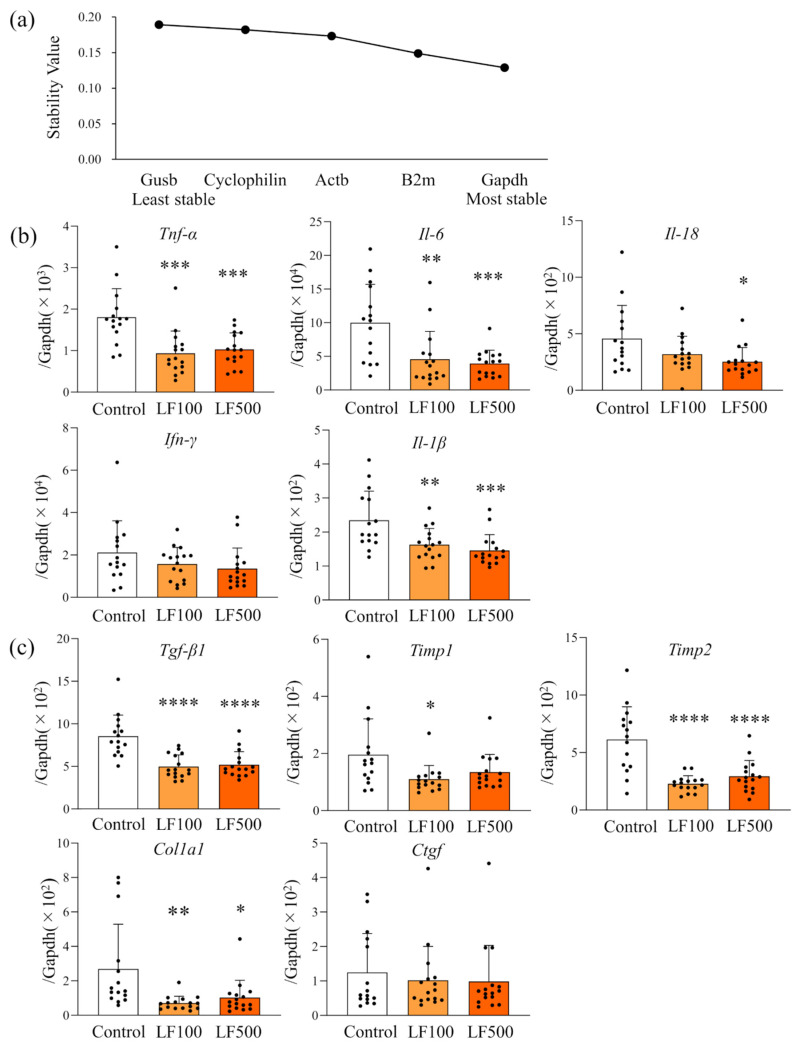
Down-regulation of inflammatory cytokines by lactoferrin in rat nonalcoholic steatohepatitis. Connexin 32 dominant negative transgenic (Cx32ΔTg) rats were fed a high-fat diet (HFD), given an intraperitoneal injection of dimethylnitrosamine (DMN), and treated with lactoferrin (LF) for 17 weeks. (**a**) NormFinder stability values for candidate housekeeping genes (*Gapdh*, *B2m*, *Actb*, *Ppia*, and *Gusb*). (**b**,**c**) mRNA levels of (**b**) pro-inflammatory cytokines (*Tnf-α*, *Il-6*, *Il-18*, *Ifn-γ*, and *Il-1β*) and (**c**) pro-fibrotic cytokines (*Tgf-β1*, *Timp1*, *Timp2*, *Col1a1*, and *Ctgf*) in Control, LF 100 mg/kg/day (LF100), or LF 500 mg/kg/day (LF500) rat groups were measured using quantitative reverse transcription (RT)–PCR. Data is shown as the mean ± SD, *n* = 15–16 per group, * *p* < 0.05, ** *p* < 0.01, *** *p* < 0.001, **** *p* < 0.0001 compared to the Control group.

Previous studies, including ours, strongly indicated that expression of inflammatory cytokines associated with inflammation *(Tnf-α*, *Il-6*, *Il-18*, *Ifn-γ*, and *Il-1β*) and fibrosis (*Tgf-β1*, *Timp1*, *Timp2*, *Col1a1*, and *Ctgf*) correlated with histological NASH activity in human and rodent models [15,26,27,28]. Thus, we further quantitated their mRNA expression level using qRT–PCR. NormFinder analysis revealed that the stability value of *Gapdh* was the smallest among examined candidate housekeeping genes (Figure 4a). Therefore, we concluded *Gapdh* as the most stable gene and used it as a reference. As shown in Figure 4b, the inflammatory cytokines, *Il-6*, *Tnf-α*, *Il-18*, and *Il-1β*, were significantly down-regulated by LF compared with the control, and a dose-dependency was observed with *Il-6*, *Il-18*, and *Il-1β*. While not significant, *Ifn-γ* mRNA expression also tended to be decreased by LF (Figure 4b and Table S2). The mRNA expression of *Tgf-β1*, *Col1a1*, *Timp1*, *Timp2*, and *Ctgf* as fibrosis-related cytokines was also measured; *Timp2*, *Col1a1*, and *Tgf-β1* were significantly down-regulated by LF (Figure 4c and Table S2). These results suggests that down-regulation of inflammatory cytokines by LF was involved in the attenuation of steatohepatitis and hepatic fibrosis in a Cx32ΔTg–HFD–DMN rat NASH model.

### 3.4. LF Administration Reduces NF-κB Signaling in Cx32ΔTg Rats

**Figure 5:**
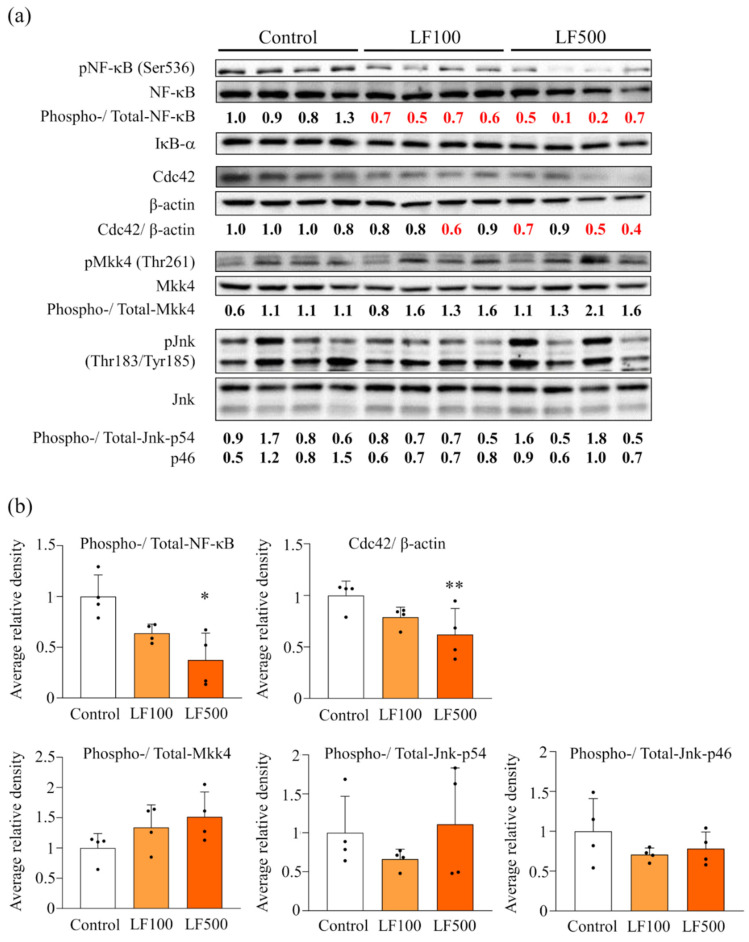
Down-regulation of inflammatory cytokines and deactivation of NF-κB and JNK signaling after the administration of lactoferrin in nonalcoholic steatohepatitis induced in Cx32 dominant negative transgenic rats. Connexin 32 dominant negative transgenic (Cx32ΔTg) rats were fed a high-fat diet (HFD), given an intraperitoneal injection of dimethylnitrosamine (DMN), and treated with lactoferrin (LF) for 17 weeks. (**a**) Protein levels of nuclear factor (NF)-κB-related (NF-κB, phosphorylated (p)NF-κB, IκB-α) and SAPK/JNK (Cdc42, Mkk4, pMkk4, Jnk, and pJnk) signaling proteins in Control, LF 100 mg/kg/day (LF100), or LF 500 mg/kg/day (LF500) rat groups were assessed by western blotting. Each lane represents a protein sample from an individual rat. Phospho, phosphorylated. (**b**) Data is shown as the mean ± SD. * *p* < 0.05, ** *p* < 0.01 compared to the Control group.

Previous studies showed that NF-κB and JNK/SAPK signaling were switched on in a rat NASH model mediated by MCDD or HFD and DMN combined [10,15]. Therefore, we investigated how such signal transduction was altered by the administration of LF. Western blotting showed that elevated pNF-κB protein expression in NASH was significantly decreased by the administration of LF in a dose-dependent manner. In contrast, phosphorylated Mkk4 and Jnk, which belong to JNK/SAPK signaling, were not affected by LF, even though their upstream protein, Cdc42, was significantly reduced in LF–treated groups (Figure 5). Such results indicate that the inactivation of NF-κB, but not JNK/SAPK signaling, is involved in the preventive effect of LF against NASH development in rats.

## 4. Discussion

In this study, we examined the chemopreventive effect of LF on NASH in a Cx32ΔTg–HFD–DMN rat NASH model. NASH is an internationally prevalent chronic liver disease that shows fatty accumulations in the liver, ballooning, and inflamed hepatocyte. Non-alcoholic fatty liver disease often occurs in adults as a complication of lifestyle-related diseases, although it has also been found in children and is increasing all over the world regardless of a country’s industrialization level [3,29]. Eventually, continuous inflammation leads to fibrosis and progresses to cirrhosis and HCC. It is known that nearly 20% of NASH cases progress to liver cirrhosis and HCC, with or without liver cirrhosis [30,31]. Therefore, NASH is one of the most crucial targets for deterring liver cirrhosis and HCC.

Currently, the main treatments for NASH include an improvement in life-style, represented by diet and exercise therapies. In addition, existing drugs for other diseases might also be effective for countering NASH. For instance, for drug repositioning, insulin sensitizers, such as pioglitazone, improved hepatocyte injury and fibrosis in a randomized, placebo-controlled trial [32]. Ratziu et al. reported that rosiglitazone decreased liver steatosis, but an improvement in hepatocyte injury and fibrosis was not found [33]. Vitamin E [34,35] and drugs for hypercholesterolemia [36] have also been used as drug therapies for NASH but have not been established as standard treatments due to their less potent medicinal effects and possible side effects after long-term use [33,37,38]. Consequently, daily intervention with functional supplements, along with lifestyle modification, is considered essential in preventing NASH progression. Considering the pathogenesis of NASH, we hypothesized that the anti-inflammatory effects of LF would prevent this disease. Previous studies proposed that LF expression in the liver was decreased in high-fructose, high-fat, or MCD-induced mouse NAFLD models [39]. In contrast, LF ameliorated HFD-induced hepatic steatosis and elevated the triglyceride level in mouse models [40,41,42]. In addition, the levels of hepatic triglycerides and visceral fat were decreased by LF and were positively correlated in ICR mice [43], indicating that LF has the potential to reduce fat accumulation in the liver.

In the present study, for the first time, the effect of LF on histological features of NASH, including steatosis, hepatocyte injury, and inflammation, was quantitated using scoring systems originally designed for humans [24,44]. Lactoferrin significantly improved steatosis in the liver but did not affect visceral fat weight in the rat NASH model. These findings indicated that LF might protect hepatic steatosis by moderating fatty acid metabolism in the liver or in adipose tissue. The balance of lipid metabolism in a whole body may determine the effect on the lipid environment in each organ. Hepatocyte injury and inflammation in NASH were also decreased in LF–treated groups. It is well-known that the inflammation-associated cytokines, *Il-6*, *Tnf-α*, and *Il-1β*, were up-regulated and involved in the evocation of chronic inflammation in the colon and liver [15,17]. As already demonstrated in the colon, down-regulation of these cytokines by LF was also induced in NASH in the present study. Altogether, LF may prevent steatohepatitis via a decrease of inflammatory cytokines.

Persistent chronic inflammation in NASH leads to increased fibrosis; similar to other chronic hepatitis diseases, the irreversible alteration of liver structure due to progressive fibrosis eventually leads to cirrhosis [45]. Therefore, fibrosis is one of the most important prognostic factors for patients with NASH. In accordance with previous studies, LF has the potential to suppress liver fibrosis induced by thioacetamide [46,47]; however, the effect on fibrosis during NASH has not yet been established. This is due to the fact that it is not easy to induce fibrosis with NASH over a short time period in an animal model. As shown in Figure 3a, advanced fibrosis when bridging between lobules, or a lobule and portal vein, was induced by LF in a Cx32ΔTg–HFD–DMN rat NASH model. Lactoferrin significantly decreased not only steatohepatitis but also the histological score and area of fibrosis in the model. The numbers of hepatic stellate cells with an active phenotype were increased in NASH and decreased in the livers of LF–treated rats. These novel findings indicate that LF prevents the liver fibrosis of NASH via the inactivation of HSC.

To date, the anti-tumor effect of LF on HCC was described in previous studies using a DEN-induced HCC model in rats or mice [48,49], but effects of LF on NASH-related hepatocarcinogenesis have not been established. The glutathione S-transferase placental form is a well-known marker for preneoplastic lesions in rat liver. Therefore, carcinogenic potentials in the liver can be measured by GST-P immunohistochemistry in the early phase of hepatocarcinogenesis [25]. The number and area of GST-P-positive foci in the liver tended to decrease in LF-treated groups, although a significant difference was not found (Figure 4). We previously induced GST-P-positive foci in a NASH model using DEN [10] or DMN [15]. Both the number and area of GST-P-positive foci induced by DMN were decreased compared to those induced by DEN, which might influence the lack of significant difference by LF in this study. The chemopreventive effect of LF on NASH-related hepatocarcinogenesis should be investigated in a future study.

Nuclear factor-κB signaling plays central roles in inflammation and fibrosis during NASH progression. Especially in regard to fibrosis, activation of NF-κB stimulates parenchymal cells, including Kupffer cells [50] and enhanced TGF-β1 signaling that is essential as a profibrogenic mediator [51]. Transforming growth factor-β1 signaling modulates HSC as an active phenotype [27,52]. However, other reports indicated that TGF-β1 induced NF-κB activation [53]. In the present study, LF treatment decreased activated HSC and prevented fibrosis in a rat NASH model. Furthermore, NF-κB activation and TGF-β1 up-regulation in the model were attenuated by LF. However, JNK, which is also an important signaling pathway for fibrosis, was not altered by LF administration. These results suggest that LF protected the development of fibrosis by inhibiting NF-κB and TGF-β1 signaling.

## 5. Conclusions

This study demonstrated that LF prevents steatohepatitis and fibrosis without any adverse effects in a Cx32ΔTg–HFD–DMN rat NASH model. Therefore, LF may be a potential preventive or therapeutic application for this disease.

## Figures and Tables

**Table 1:** Sequence of primers for housekeeping genes tested with quantitative reverse transcription PCR.

Symbol	Gene Name	Accession Number	Primers (5′-3′)
Gapdh	Glyceraldehyde-3-phosphate dehydrogenase	NM_017008.4	GCATCCTGCACCACCAACTG GCCTGCTTCACCACCTTCTT
B2m	Beta-2 microglobulin	NM_012512.2	CCTTCAGCAAGGACTGGTCT
			TACATGTCTCGGTCCCAGGT
Actb	Actin, beta	NM_031144.3	GCGAGTACAACCTTCTTGCAG
			CATACCCACCATCACACCCTG
Ppia	Peptidylprolyl isomerase A	NM_017101.1	TGCTGGACCAAACACAATG GAAGGGGAATGAGGAAAATA
Gusb	Glucuronidase, beta	NM_017015.3	CCGACAGGAGAGTGGTGTTG GCTTGGTGATGTCAGCCTCA

**Table 2:** Body and various organ weights in connexin 32 dominant negative transgenic rats fed a high-fat diet and dimethylnitrosamine with or without lactoferrin (100 or 500 mg/kg/day) at week 17.

	No. of rats	Body Weight (g)	Liver		Kidney		Visceral Fat	
			Absolute (g)	Relative (%)	Absolute (g)	Relative (%)	Absolute (g)	Relative (%)
Control	15	564.7 ± 67.9	12.22 ± 2.70	2.14 ± 0.33	2.59 ± 0.15	0.48 ± 0.09	15.34 ± 4.68	2.65 ± 0.64
LF100	16	607.9 ± 63.2	14.59 ± 1.91 **	2.40 ± 0.18 *	2.67 ± 0.16	0.44 ± 0.04	16.90 ± 4.56	2.74 ± 0.53
LF500	16	585.8 ± 33.3	13.37 ± 1.79	2.28 ± 0.22	2.66 ± 0.18	0.46 ± 0.03	14.98 ± 3.15	2.54 ± 0.44

LF100, lactoferrin 100 mg/kg/day; LF500, lactoferrin 500 mg/kg/day. Dunnett’s test *: *p* < 0.05, **: *p* < 0.01 vs. Control.

**Table 3:** Hepatic enzyme serum levels in connexin 32 dominant negative transgenic rats fed a high-fat diet and dimethylnitrosamine with or without lactoferrin (100 or 500 mg/kg/day) at week 17.

	No. of rats	TP (g/dL)	ALB (g/dL)	AST (U/L)	ALT (U/L)	ALP (U/L)	GLU (mg/dL)	T-chol (mg/dL)	LDL-C (mg/dL)	HDL-C (mg/dL)
Control	15	5.9 ± 0.6	4.1 ± 0.2	117.0 ± 86.6	45.5 ± 11.8	1249.9 ± 516.2	136.0 ± 18.2	91.1 ± 68.9	16.9 ± 15.0	46.8 ± 11.4
LF100	16	6.1 ± 0.2	4.1 ± 0.2	88.9 ± 21.5	47.4 ± 14.6	922.6 ± 286.9	161.2 ± 29.2 *	74.4 ± 14.4	10.6 ± 2.7	51.6 ± 11.4
LF500	16	6.0 ± 0.3	4.1 ± 0.2	91.2 ± 21.0	45.4 ± 9.7	1061.7 ± 437.1	153.0 ± 33.0	72.2 ± 13.3	11.1 ± 2.9	48.6 ± 9.3

Alb, albumin; ALP, alkaline phosphatase; ALT, alanine aminotransferase; GLU, glucose; HDL-C, high-density lipoprotein cholesterol; LDL-C, low-density lipoprotein cholesterol; LF100, lactoferrin 100 mg/kg/day; LF500, lactoferrin 500 mg/kg/day; T-chol, total cholesterol; TP, total protein. Dunnett’s test *: *p* < 0.05 vs. Control.

## Data Availability

The data presented in this study are available on request from the corresponding author.

## Supplementary Materials

The following supporting information can be downloaded at: https://www.mdpi.com/article/10.3390/nu14010042/s1, Table S1: Histopathology of NASH, fibrosis, and hepatocarcinogenesis in connexin 32 dominant negative transgenic rats fed a high-fat diet and dimethylnitrosamine with or without lactoferrin (100 or 500 mg/kg/day) at week 17; Table S2: mRNA level of inflammatory cytokines using quantitative reverse transcription PCR.

## Abbreviations

The following abbreviations are used in this manuscript:

Alb	Albumin
AST	Aspartate aminotransferase
Bex1	Brain expressed, X-linked 1
Cx	Connexin
Cx32ΔTg	Cx32 dominant negative transgenic
DEN	Diethylnitrosamine
DMN	Dimethylnitrosamine
GST-P	Glutathione S-transferase placental form
HCC	Hepatocellular carcinoma
HFD	High-fat diet
HSC	Hepatic stellate cell
IL	Interleukin
LDL-C	Low-density lipoprotein cholesterol
LF	Lactoferrin
MCDD	Methionine choline-deficient diet
NAFLD	Non-alcoholic fatty liver disease
NAS	Non-alcoholic fatty liver disease activity score
NASH	Non-alcoholic steatohepatitis
NF-κB	Nuclear factor-κB
ROS	Reactive oxygen species
TGF	Transforming growth factor
TNF	Tumor necrosis factor
Wt	Wild-type
