# Supplementary material for: Lactoferrin Prevents Hepatic Injury and Fibrosis via the Inhibition of NF-κB Signaling in a Rat Non-Alcoholic Steatohepatitis Model
Source: Nutrients. 2021 Dec 23;14(1):42. doi: 10.3390/nu14010042 (PMC8746867; doi:10.3390/nu14010042)
Supplement: Supplementary file 1 [file nutrients-14-00042-s001.zip › nutrients-1492645-supplementary.pdf]

Table S1. Histopathology of NASH, fibrosis and hepatocarcinogenesis in connexin 32 dominant negative transgenic rats fed a high fat diet and dimethylnitrosamine with or without lactoferrin (100 or 500 mg/kg/day) at week 17.

	Score					Azan (%)	α-SMA (%)	GST-P Number (/cm²)	GST-P Area (mm²/cm²)
	Steatosis	Lobular inflammation	Ballooning	NAS	Fibrosis				
Control	3.0 ± 0.0	2.3 ± 0.4	1.9 ± 0.3	7.1 ± 0.5	3.3 ± 0.5	12.9 ± 8.4	6.3 ± 4.4	20.9 ± 11.9	0.51 ± 0.54
LF100	2.8 ± 0.4	1.4 ± 0.5****	1.3 ± 0.4***	5.4 ± 0.9****	2.6 ± 0.5***	8.1 ± 4.0****	3.4 ± 3.3****	17.4 ± 9.6	0.36 ± 0.60
LF500	2.4 ± 0.6**	1.2 ± 0.4****	1.1 ± 0.3****	4.8 ± 0.8****	2.4 ± 0.5****	7.2 ± 5.0****	2.1 ± 1.5****	16.8 ± 5.7	0.32 ± 0.32

α-SMA, α-smooth muscle actin; GST-P, glutathione S-transferase placental form; LF100, lactoferrin 100 mg/kg/day; LF500, lactoferrin 500 mg/kg/day; NAS, non-alcoholic fatty liver disease activity score; NASH, non-alcoholic steatohepatitis

Dunnett's test *: P<0.05, **: P<0.01, ***: P<0.001, ****: P<0.0001 vs. Control

Table S2. mRNA level of inflammatory cytokines using quantitative reverse transcription PCR.

	Tnf-α	***Il-6***	***Il-18***	***Ifn-γ***	***Il-1β***	***Tgf-β1***	***Timp1***	***Timp2***	***Col1a1***	***Ctgf***
Control	1.8 ± 0.7	10.0 ± 5.7	4.6 ± 3.0	1.8 ± 0.9	2.3 ± 0.9	8.5 ± 2.5	2.0 ± 1.3	6.1 ± 2.8	2.7 ± 2.6	1.2 ± 1.1
LF100	0.9 ± 0.5***	4.6 ± 4.1**	3.2 ± 1.6	1.4 ± 0.7	1.6 ± 0.5**	5.0 ± 1.4****	1.1 ± 0.5*	2.3 ± 0.7****	0.7 ± 0.4**	1.0 ± 1.0
LF500	1.0 ± 0.4***	3.9 ± 2.0***	2.5 ± 1.3*	1.6 ± 1.5	1.5 ± 0.5***	5.2 ± 1.5****	1.3 ± 0.6	2.9 ± 1.4****	1.0 ± 1.0*	1.0 ± 1.0

Col1a1, collagen1a1; Ctgf, connective tissue growth factor; Ifn-γ, interferon-γ; Il-1β, interleukin-1β; Il-6, interleukin-6; Il-18, interleukin-18; LF100, lactoferrin 100 mg/kg/day; LF500, lactoferrin 500 mg/kg/day; Tgf-β1, transforming growth factor-β; Timp1, tissue inhibitor of metalloproteinase-1; Timp2, tissue inhibitor of metalloproteinase-2; Tnf-α, tumor necrosis factor-α

Dunnett's test *: $P<0.05$, **: $P<0.01$, ***: $P<0.001$, ****: $P<0.0001$ vs. Control
